# Fungus Hæmatodes

**Published:** 1836

**Authors:** 


### Art. IV.—Fungus Hæmatodes. *By M. Smith, of North Carolina.*

In the American Journal of the Medical Sciences for May last, we have an interesting report of a case of Fungus Hæmatodes of the Eye, which was entirely removed without any return of the disease, although the operation was performed more than a year since. The subject was a colored boy, ten years old, of good constitution and without any hereditary taint of the kind whatever, although the affection seemed to commence in a white spot, which gradually enlarged into an extensive tumor, when the child was only six months old. The eye was extirpated in the usual manner, and, although there was "*furious* hæmorrhage" in the early stage of the operation, the discharge was speedily arrested by moderate pressure after the tumor was removed. When examined, both before and after the operation, no trace of the healthy structure of the eye was apparent. The socket was kept filled with lint, under a moderate pressure, until granulation ceased and the cavity was quite filled. The operator attributes much of his success to the continued pressure he employed, notwithstanding Mr. Lawrence disapproves of the process. The diet of the boy for a short time before, and constantly after the operation until he escaped from the hands of the surgeon, was corn bread and mush, of the salutary effects of which the reporter entertains a high opinion.

Although the operator seems to entertain no doubt as to the nature of the tumor, still we are not decided, even from his own description, that it was a true fungus hæmatodes. The medullary structure, so characteristic of the disease, was not present. It is true, the writer informs us that the tumor "consisted of that vascular or fibro-vascular network, denominated *accidental erectile tissue*, or *fungus hæmatodes*, strictly speaking," yet when he describes its intimate structure he informs us that it exhibited generally the appearance of areolar tissue. Indeed, the whole description, especially when taken in connection with the fact that it did not reappear after the operation, convinces us that it was not connected with a constitutional taint; and, therefore, it cannot be considered as a true fungus hæmatodes. The great variety of tumors approximating malignant fungus formations, especially of the eye, are often so vaguely described by authors, that it is difficult to determine the precise nature of the disease. As the reporter, however, seems to be a close observer, as well as a good scholar and correct writer, we would be willing to concede much to his opinion, especially as we have not been able to examine for ourselves.

In connection with the above case, Mr. Smith gives us the description of an encepaloid tumor, taken from a female breast, a few weeks previous to his report, but he does not state whether his patient survived the operation any time or not. W. W.

